# Efficacy of a theoretical-practical course for the ultrasound measurement of the optic nerve diameter in different healthcare operators

**DOI:** 10.1186/s13089-025-00431-7

**Published:** 2025-06-16

**Authors:** Eugenio Garofalo, Giuseppe Neri, Vincenzo Bosco, Zaninni Caroleo, Fabiola Virdò, Helenia Mastrangelo, Giusy Guzzi, Gianmaria Cammarota, Chiara Robba, Federico Longhini, Andrea Bruni, ONSD study group, Aldo Mesiti, Antonio Camastra, Antonio Caroleo, Daniele Commisso, Arianna Conidi, Anna Maria Froio, Giuseppe Gaetano, Giuseppe Guerriero, Lucia Lentini, Giuseppe Mazza, Federica Mellace, Silvia Riillo, Giusy Ruocco, Giuseppe Saraco, Deborah Veltri

**Affiliations:** 1 Anesthesia and Intensive Care, Department of Medical and Surgical Sciences, “Magna Graecia” University, Catanzaro, Italy; 2 Department of Neurosurgery, “R Dulbecco” University Hospital, Catanzaro, Italy; 3 Department of Translational Medicine, Eastern Piedmont University, Novara, Italy; 4 Department of Surgical Science and Integrated Diagnostic, University of Genova, Genoa, Italy; 5 Anesthesia and Intensive Care, IRCCS for Oncology and Neuroscience, Policlinico San Martino, Genoa, Italy; 6 Intensive Care Unit, “Mater Domini” University Hospital, Department of Medical and Surgical Sciences, Magna Graecia University, Viale Europa, 88100 Catanzaro, Italy

**Keywords:** Optic nerve sheath diameter, Training, Nurse, Student, Intracranial pressure, Ultrasound, Intensive care unit, Accuracy

## Abstract

**Background:**

The measurement of the optic nerve sheath diameter (ONSD) via ultrasound is a non-invasive technique to estimate intracranial pressure. Brief training has been shown to be effective in accurately teaching the ONSD technique in specialized healthcare providers. This study evaluates the ability of medical and nursing students, Intensive Care Unit (ICU) nurses, and ICU residents to perform ONSD measurements after a brief training.

**Methods:**

Forty participants underwent a 4-h training session consisting of 30 min of lecture focusing on the key principles of the technique for ONSD measurement, followed by at least 20 measurements with an expert tutor. Thereafter, all participants assessed 5 ONSD measurements on healthy volunteers and their assessments were compared to those by the expert tutor.

**Results:**

All participants successfully visualized the optic nerve and recorded similar values among groups (p > 0.05 for all comparisons). ICU nurse residents and medical students demonstrated a good accuracyof measurements, as defined by an upper and lower limits of agreement with the expert tutor not exceeding 0.5 mm in the Bland–Altman analysis. On the opposite, nurse students had the highest error rates and poor accuracy in ONSD assessment.

**Conclusions:**

These findings highlight the feasibility of training medical students, ICU nurses and residents in ONSD measurement, opening the possibility of a wider application of this technique. After dedicated training, ONSD assessment and monitoring could be performed not only by specialists but also by other healthcare providers, including specialized nurses. This may serve as an additional tool for the rapid triage of patients, even in out-of-hospital settings.

**Supplementary Information:**

The online version contains supplementary material available at 10.1186/s13089-025-00431-7.

## Introduction

The measurement of the optic nerve sheath diameter (ONSD) is a non-invasive diagnostic technique used to estimate intracranial pressure (ICP). The optic nerve sheath, which surrounds the optic nerve, is continuous with the subarachnoid space, which contains cerebrospinal fluid [1]. When ICP increases, the pressure in cerebrospinal fluid in the optic nerve sheath also increases, causing an enlargement of its elastic diameter [2].

The measurement of ONSD is generally performed by placing a linear ultrasound probe on the closed eyelid to visualize the optic nerve behind the eyeball [3]. Guidelines on how to measure ONSD have recently been published in a statement by an international consensus of experts [3].

The correlation between increased ONSD and elevated ICP is well documented [2, 4]. Although it is not a continuous monitoring method, ONSD measurement can be quickly and repeatedly performed at the patient’s bedside even in remote locations without causing significant discomfort to the patient, which may be invaluable for patient triage and management. It can also be useful for the assessment of critically ill patients in tertiary care centres, especially those too unstable to be transported to the radiology department for a Computed Tomography (CT) scan or Magnetic Resonance Imaging (MRI) [5].

The diagnostic cut-off values indicating elevated ICP vary across studies and meta-analyses. Generally, reported values range between 4.8 and 6.4 mm [6, 7]. This variability is influenced by several factors, including measurement techniques, equipment used, and operator experience [5, 8–10]. Ballantyne et al. conducted a study in which an experienced pediatric radiologist and two radiology residents measured ONSD in a group of adult patients. After examining 17 patients, they reviewed their technique and subsequently examined another 50 patients, observing a significant improvement in measurement accuracy [11]. Potgieter et al. demonstrated that beginner operators could be trained in a single supervised 4-h training session to perform ultrasound measurements of ONSD in healthy volunteers. Their findings showed clinically acceptable accuracy comparable to that of an experienced sonographer, indicating that the technique is easy to learn and reproducible among different and mixed specialized operators, although adequate but brief training is necessary [5]. A recent consensus of the Neurocritical Care Society has defined the ONSD assessment as competence with a basic-plus skill among all brain ultrasonography technique to be applied in acute brain injured patients [12]. Indeed, this competence could be acquired by specialists after a dedicated training program and around 20 examinations [13]. 

To date, no clear and direct data demonstrate that healthcare professionals other than medical specialists (with varying levels of expertise) can learn this technique and obtain reliable ONSD measurements. Only Potgieter et al. showed that two registered nurses, who had never operated an ultrasound probe prior the study, did not guarantee an acceptable measure of the ONSD [5]. Therefore, we designed this study to assess the ability of nursing and medical students, Intensive Care Unit (ICU) nurses and residents, to visualize and correctly measure ONSD in healthy volunteers after a brief training on the technique.

## Materials and methods

This prospective observational study was conducted in the classrooms of the “Magna Graecia” University of Catanzaro (Italy) after approval by the local ethics committee (approval number 373/2024 on 3rd January 2025). Written informed consent was obtained from all participants. The trial was prospectively registered on clinicaltrials.gov (record number NCT06830460) and it follows the STrengthening the Reporting of OBservational studies in Epidemiology (STROBE) guidelines for observational studies [14].

### Subjects

A total of 40 volunteer participants naive to the ONSD measurement technique were recruited as follows: 10 medical students at their last year of training, ten nursing students at their last year of training, ten nurses with at least 3 years of experience in ICU and ten ICU residents. Volunteers were recruited through an internal communication in the ICU department and in the academic classes of our university. During the communication, the study protocol was exposed, and volunteer participants were therefore recruited at the end of the presentation. All subjects underwent a training and a verification session.

### Training session

All participants attended a theoretical-practical training session of approximately 4 h conducted by an expert physician, focusing on the key principles of the technique, including acoustic windows and anatomical landmarks for ONSD measurement [3], following the training proposed by Potgieter et al. [5].

In particular, the session included a 30-min lecture covering the anatomy of the eyeball and orbit, ultrasound technique, and ONSD measurement, followed by a real-time demonstration of how to obtain ONSD measurements. Within the training sessions, participants performed at least 20 supervised ONSD measurements under the guidance of an expert tutor to familiarize themselves with the ultrasound procedure and reduce intra- and inter-observer variability, as suggested by recent literature [3, 5, 10]. At the end of the session, each participant had the opportunity to perform five additional examinations if they did not yet feel confident with the technique [3, 5, 10].

### Verification session

All participants took a multiple-choice questionnaire consisting of 10 questions. A score of at least 70% was required to pass [15]. Those who passed the theoretical test proceeded to the practical assessment. In the practical assessment, each participant performed ONSD measurements on five healthy volunteers who were not involved in the training session. These measurements were recorded and compared to the reference measurement performed by the expert tutor (F.L.). Of note, the expert tutor measured the internal ONSD independently and prior to the measurements performed by the trainees.

The verification session was conducted immediately after the training session to prevent participants from gaining additional practice in between, to avoid potential bias. ICU nurses and residents might have had opportunities to further train during their clinical duties, whereas medical and nursing students would not, potentially creating a significant bias between groups. Therefore, the study was structured to proceed with the verification session directly following the training session.

### ONSD measurement technique

The technique presented to trainees and subsequently applied for measurement followed the recent expert consensus on quality criteria for ONSD evaluation [3]. Operators used an ultrasound machine equipped with an ocular ultrasound present and a linear probe with a minimum effective frequency of 7.5 MHz (MyLabTM30, Esaote, Genova, Italy).

Healthy volunteers were positioned semi-seated with a 45° head elevation, maintaining a neutral gaze with closed eyelids. The image was acquired by centring the eyeball within the frame in an axial plane and adjusting the ultrasound beam to parallelly insonate the optic nerve and to take perpendicularly the measure. Once the image was sufficiently clear to distinguish anatomical structures without artifacts, it was captured for ONSD measurement. The measurement was taken 3 mm posterior to the retina, considering the internal ONSD [3]. Both the training and verification sessions were conducted on the same healthy volunteers, who had no neurological comorbidities, for all 40 participants. Only the right ONSD was measured, as all subjects were healthy.

The obtained measurements were recorded by a researcher not involved in the ONSD training or assessment. Throughout all scans, operators were reminded to avoid applying pressure to the eyeball using sufficient ultrasound gel and supporting the hand on the patient’s forehead, nasal bridge, or cheek [3]. Additionally, volunteers were repeatedly asked to report any discomfort or pressure beyond a gentle touch on the eyes during each measurement. This ensured both safety and measurement accuracy, as excessive pressure on the eyeball could cause eye injury, increase intraocular pressure, oculocephalic (trigeminovagal) reflex, bradycardia and hypotension [16]. The probe was properly cleaned and disinfected between each measurement.

Throughout the entire measurement procedure, the expert tutor recorded any execution errors using a specially designed checklist (see the Electronic Supplemental Materials). Specifically, the expert assessed whether: (1) the trainees correctly positioned the probe on the closed upper eyelid, (2) the globe was centred in the image, (3) the transverse axis was properly aligned, (4) the ultrasound projection was appropriately adjusted, ((5) the optic nerve was positioned at 90°, 6) artifacts were absent or the operator recognized artefacts and improved the quality of the imaging, (7) the measurement was taken perpendicularly, (8) the measurement was performed 3 mm posterior to the retina, (9) the internal ONSD was correctly assessed, and (10) the healthy volunteers reported any sensation of pressure on the eye [3].

From the recorded ONSD values, we also calculated the difference between the trainee’s assessment and the expert’s evaluation (ΔONSD) to assess the inter-observer variability per group in comparison to the expert sonographer. Within each trainee group, we also analysed the ΔONSD values to exclude a time-dependent improvement of measurements ability during the verification session.

### Statistical analysis

In keeping with similar previous studies [5, 10, 17], we enrolled 10 volunteers per group of healthcare operators with different skills.

The data are presented in a non-parametric manner [i.e., as the median (interquartile range)], considering the relatively small number of participants per group. Continuous variables were compared using Friedman’s repeated-measures analysis of variance on ranks or the Kruskal–Wallis test for non-repeated measures. Post-hoc multiple comparisons were conducted using Dunn’s method when comparing data against a single control group (i.e., the expert) or Tukey’s multiple comparisons test when comparing all groups with each other.

We also determined the correlation coefficients (ρ) [95% CI] between the measurements obtained by the expert and trainees in each group, along with the corresponding p-values, using Spearman’s rank correlation test.

Finally, the Bland–Altman plot was used to assess the agreement between the measurements performed by the expert (gold standard) and every single group of participants, as per level of skills. Specifically, we separately plotted the mean differences between the measurements recorded in the four groups and those recorded by the expert and the upper and lower limits of agreement [95% confidence of interval], calculated as the mean + 1.96 × standard deviation (1.96 × SD) and mean − 1.96 × SD, respectively. Based on previous studies by Potgieter et al. [5] and Ballantyne et al. [11], a value of upper and lower limits of agreement not exceeding 0.5 mm were defined as the acceptable limit for practical purposes in evaluating the width of the plot and, consequently, the acceptability of the performed measurements. P-values < 0.05 were considered statistically significant. Statistical analysis was performed using the Sigmaplot v. 12.0 (Systat Software Inc., San Jose, California).

## Results

Forty participants participated in the study. In each group, gender balance was maintained, with an equal 50% representation of males and females.

All participants completed the training session. All individuals passed the multiple-choice questionnaire test (score 92.3 ± 7.7%) accessing to the second phase of the study regarding evaluation of the ability and accuracy of ONSD assessment. In particular, the percentages (median [interquartile range]) of correct answers in questionnaire were 85 [80; 90]% among nurse students, 90 [90; 100]% among ICU nurses, 90 [90; 100]% among medical students and 100 [90; 100]% among ICU residents.

Among the training participants, medical and nursing students had no prior ultrasonography experience, whereas ICU nurses had experience only in peripheral vascular cannulation and urinary bladder evaluation. ICU residents, on the other hand, were competent in ultrasound-guided vascular cannulation, peripheral nerve blocks, as well as diaphragm and lung ultrasonography. None of them had experience in brain ultrasonography techniques, including transcranial Doppler.

### ONSD measurements

All participants successfully obtained a sonographic image of the optic nerve.

The execution error rates identified by the expert were 16 [11; 22]% in the nurse students group, 9 [6; 13]% among ICU nurses, 2 [2; 4]% among medical students, and 0 [0; 3]% among ICU residents (p < 0.001).In particular, nurse students made more errors compared to ICU nurses (p = 0.011), medical students, and ICU residents (p < 0.001 for both). Additionally, ICU nurses had a higher error rate compared to medical students (p = 0.018) and ICU residents (p = 0.002). Interestingly, the error rates between medical students and ICU residents were similar (p = 0.865).

The five measurements taken by the expert and each group of trainees are presented in Table 1. Although variance was observed in two out of the five measurements, no significant differences were found between the trainees' groups and the expert’s values.

**Table 1:** ONSD values (mm) recorded by the expert and trainees

ID_Measure	Expert	Nursing Students (n = 10)	ICU Nurses (n = 10)	Medical Students (n = 10)	ICU Residents (n = 10)	Friedman P value	Dunn’s P values
#1	4.2	4.4 [4.2; 4.6]	4.3 [4.1; 4.3]	4.2 [4.1; 4.3]	4.3 [4.2; 4.3]	0.073	n.a
#2	4.0	3.9 [3.7; 4.2]	4.1 [4.0; 4.2]	4.1 [3.9; 4.2]	4.0 [3.8; 4.1]	0.142	n.a
#3	3.8	3.7 [3.6; 3.9]	3.7 [3.8; 3.8]	4.0 [3.8; 4.1]	3.9 [3.7; 3.9]	0.027	p > 0.05 for all comparisons
#4	4.0	4.0 [3.8; 4.1]	4.1 [4.0; 4.2]	4.1 [4.0; 4.2]	4.0 [3.9; 4.2]	0.043	p > 0.05 for all comparisons
#5	3.8	3.6 [3.4; 3.8]	3.8 [3.8; 3.9]	3.8 [3.5; 3.9]	3.9 [3.8; 4.0]	0.201	n.a

Data are expressed in millimeters as median [25th; 75th percentiles]

*ICU* Intensive care unit, *n.a.* not applicable

In addition, the measures obtained by all groups of trainees moderately correlated with those by the expert. In particular, the correlation coefficient (ρ) [95% CI] were 0.62 [0.41 to 0.76] (p < 0.001) in nurse students, 0.64 [0.44 to 0.78] (p < 0.001) in ICU nurse, 0.55 [0.32 to 0.72] (p < 0.001) in medical students and 0.56 [0.33 to 0.72] (p < 0.001) in ICU residents.

Table 2 displays the median [interquartile range] of the ΔONSD values in all trainees’ groups for the five verification measurements. Table 2 also reports that ΔONSD values did not decrease within all groups during the verification phase (all p values > 0.05), excluding a time-dependent improvement of measurements ability during the verification session and confirming that the training curve had already plateaued.

**Table 2:** ΔONSD values (mm) recorded in the trainees

Trainees	#1	#2	#3	#4	#5	Friedman P value
Nursing students (n = 10)	0.20 [− 0.05; 0.40]	− 0.10 [− 0.33; 0.05]	− 0.10 [− 0.23; 0.05]	− 0.20 [− 0.05; 0.05]	− 0.20 [− 0.40; 0.10]	0.120
ICU nurses (n = 10)	0.10 [− 0.15; 0.10]	0.10 [0.00; 0.23]	0.00 [− 0.13; 0.03]	0.10 [− 0.03; 0.20]	0.00 [0.00; 0.15]	0.260
Medical students (n = 10)	− 0.05 [− 0.13; 0.13]	0.10 [− 0.10; 0.23]	0.20 [0.00; 0.30]	0.10 [0.00; 0.23]	0.00 [0.30; 0.13]	0.130
ICU residents (n = 10)	0.10 [0.00; 0.13]	0.05 [− 0.18; 0.13]	− 0.10 [0.05; 0.13]	0.00 [− 0.13; 0.20]	0.10 [− 0.03; 0.20]	0.620

Data are expressed in millimeters as median [25th; 75th percentiles]

*ICU* Intensive Care Unit

**Fig. 1:**
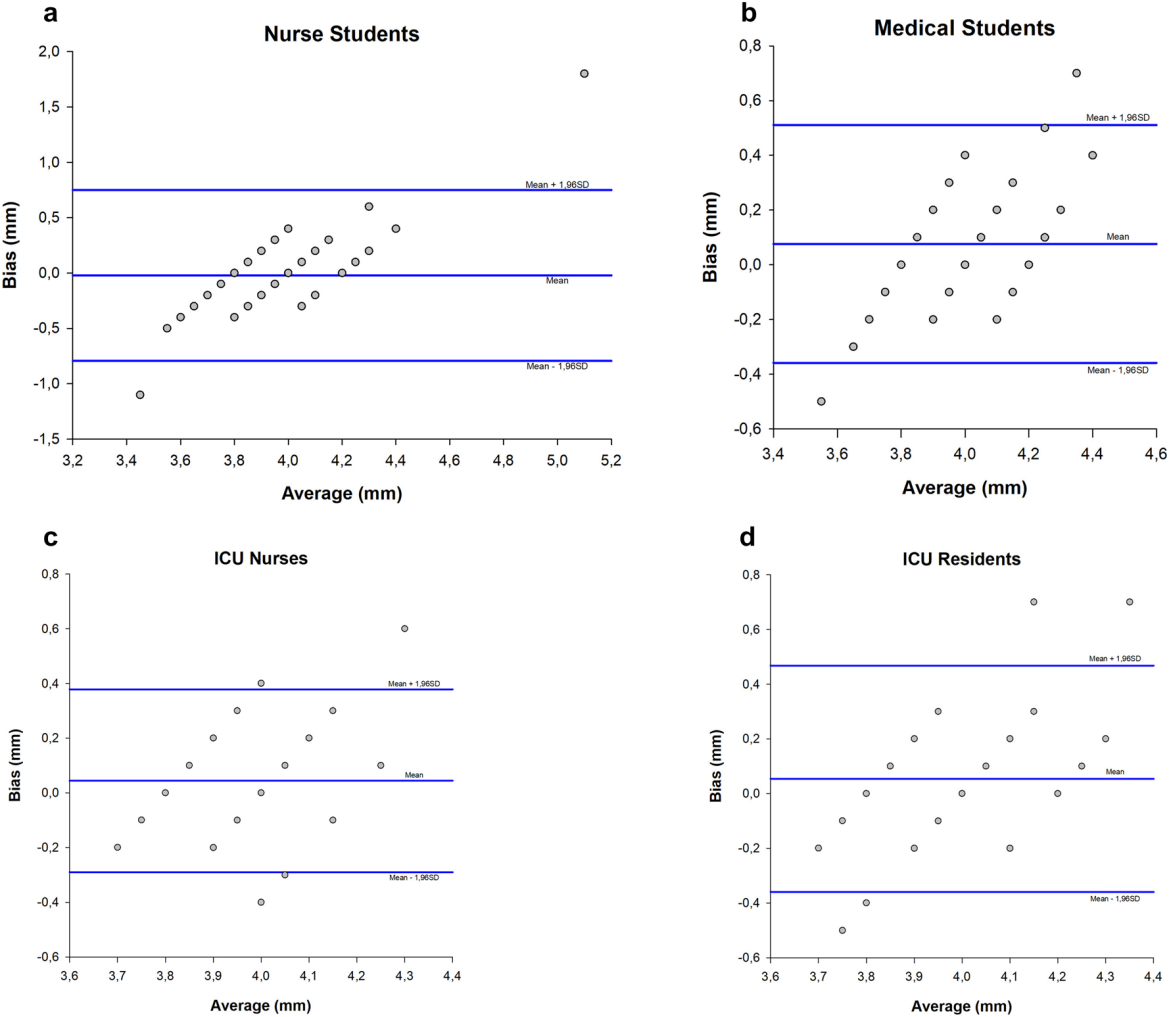
Bland–Altman Graphs. Bland–Altman plot for ONSD values by the expert compared to trainees are depicted for** a**. nurse students,** b**. medical students,** c**. ICU nurses and** d**. ICU residents. The horizontal line in the middle represents the bias of error, whereas the lines on the top and the bottom the upper and lower limits of agreement

Table 3 presents the mean [95% CI] bias, standard deviation, and the lower and upper limits of agreement between each trainee group and the expert measurements, as assessed by Bland–Altman analysis. All groups, except for nurse students, provided acceptable ONSD measurements, as the computed upper and lower limits of agreement did not exceed 0.5 mm, in accordance with our predefined criteria. Bland–Altman graphs are report in Fig. 1, separately for each trainee group.

**Table 3:** Bland–Altman graph analysis

Group	Bias	Standard Deviation	Lower limit of agreement	Upper limit of agreement
Nurse students	− 0.220 [− 0.134 to 0.090]	0.394	− 0.794 [− 0.988 to − 0.600]	0.750 [0.556 to 0.944]
ICU nurses	0.044 [− 0.004 to 0.092]	0.170	− 0.290 [− 0.374 to − 0.206]	0.378 [0.294 to 0.462]
Medical students	0.076 [0.013 to 0.139]	0.222	− 0.359 [− 0.468 to − 0.250]	0.511 [0.402 to 0.620]
ICU residents	0.054 [− 0.006 to 0.114]	0.211	− 0.360 [− 0.464 to − 0.256]	0.468 [0.364 to 0.572]

Data are expressed in millimeters

## Discussion

Our study demonstrates that not all healthcare operators can be trained in a single, supervised training session to measure ONSD in healthy volunteers to obtain a similar level of skills.

We decided to use the training method used by Potgieter et al. [5], which was already validated. A previous study demonstrated that a short training session enabled five novices (an ICU senior medical officer, a junior ICU registrar, a junior neurosurgical trainee, and two registered nurses) to acquire adequate skills in ONSD assessment in healthy volunteers [5]. However, the two registered nurses, who had never used an ultrasound probe prior the study, were unable to obtain an acceptable measure of the ONSD [5]. Therefore, given that highly specialized physicians could accurately learn how to measure the ONSD, we aimed to assess whether healthcare operators with different skill levels, from lower (nursing students) to higher (ICU residents), would visualize and measure ONSD differently in healthy volunteers after undergoing the same training [5].

The novelty of our study relies also in the demonstration that medical students, ICU residents and nurses may acquire a confident ability in assessing the ONSD, whereas nursing students do not. This topic is particularly relevant in the pre-hospital settings. Emergency Medical Services (EMS) play a crucial role in delivering advanced medical care in different contexts, especially in out of hospital in urgent and emergency situations where invasive intracranial pressure is not available/indicated. With the growing use of US machines on ambulance services, improving the use of ONSD ultrasonography may be essential to ensure efficiency, patient safety, and minimal response time from the initial alarm to arrival at the scene [18]. In some countries, single-responder units have been introduced following telephone triage. These units, staffed by highly experienced registered nurses working alone, serve as a specialized response system [19–21]. Their primary role is to assess patients, determine the appropriate level of care, decide on the necessity of ambulance transport, and initiate urgent or life-saving interventions while awaiting ambulance arrival [19–21]. Nurses or physicians in pre-hospital settings can potentially assess elevated ICP [22–24], aiding in the decision of the most appropriate hospital for patient transport. This tool may be also especially relevant in low- and middle-income countries, as well as in austere, rural, and remote settings, where invasive tools are not available [25, 26]. However, it is important to emphasize that our results are based on measurements performed in healthy volunteers, which inherently limits their direct translation into clinical practice. In real-world settings, the assessment of ONSD in unconscious or uncooperative patients presents greater challenges, primarily due to difficulties in maintaining a straight gaze, which increases both the technical complexity and the reliance on operator skill. Furthermore, the presence of enlarged ONSD values, as seen in pathological conditions, can amplify measurement errors, especially when image acquisition is suboptimal.

The importance of ONSD assessment was recently emphasized in the ‘Brussels Consensus for Non-Invasive ICP Monitoring’ for cases where invasive systems are unavailable in the management of traumatic brain injury [26]. ONSD has been recognized among non-invasive techniques and is recommended for detecting elevated ICP. When high-quality images are obtained, elevated ONSD values together with another noninvasive tool (pupillometry, transcranial Doppler) may guide treatment decisions [26]. Additionally, ONSD can serve as a non-invasive monitoring tool, repeated every 4–6 h in patients with moderate traumatic brain injury (Glasgow Coma Scale 9–12). However, it is important to highlight that in severe traumatic brain injury (Glasgow Coma Scale < 9) with radiological signs of intracranial hypertension, invasive ICP monitoring-if available-remains the preferred approach [26].

In our analysis we also aimed to confirm that our groups were able to reach accuracy in performing the ONSD assessments at their plateau in the learning curves. The learning curve for point-of-care ultrasound has been explored in numerous studies across various techniques, ranging from 10 [27, 28] to 200 scans [29]. When focusing on the ONSD learning curve, Tayal et al. concluded that this technique can be acquired relatively quickly, requiring between 10 and 25 scans depending on prior ultrasound experience [30]. With a properly designed study and data analysis, Zeiler et al. defined that 21 examinations are required to reach the plateau in the learning curve for ONSD assessment [13]. These findings may help explain our results, as our trainees completed at least 20 ONSD measurements before participating in the verification session.

In keeping also with previous data [5, 11, 31], the ΔONSD values between our groups of trainees and the expert are restrained to small values, that translated into a good agreement of measures only for ICU nurses and residents and medical students. Our predetermined lower and upper limit of agreement (< 0.5 mm) has been set based on the findings in previous studies [5, 11].

Similarly to our results, another study conducted in a different setting (i.e. 23 military trainees with minimal prior experience in point-of-care ultrasound) demonstrated the ONSD can be learned quickly, with an accuracy comparable with ultrasound experts [32]. Indeed, the bias values reported in the Bland–Altmann analysis are very similar to ours in the groups of medical students, ICU nurses and residents.

Before drawing our conclusions, several limitations must be acknowledged. First, our findings are based on a single-centre study, requiring further confirmation and validation in different settings, with various ultrasonography machines, and on actual patients rather than healthy volunteers. Additionally, the expertise of the instructor providing the training may have influenced the results.

Second, our study included only 10 volunteers per group, raising the possibility of selection bias. However, to mitigate this issue, we strictly predefined the inclusion criteria for trainees. In addition, each trainee performed only five ONSD assessments during the verification session. This limited number of assessments was intentionally chosen to ensure that both the training and verification sessions could be completed within a single day for all participants. This approach aimed to further minimize the risk of bias related to potential practice opportunities between sessions (as discussed below) and to reduce variability in ONSD measurements that could occur if assessments were conducted on different days in healthy volunteers. However, as shown in Table 2, the ΔONSD values across the five assessments were not significantly different among the groups, demonstrating, as mentioned above, that participants had already reached a plateau in their learning curves, in line with findings reported in the literature [13, 30]. Therefore, additional measurements would likely have only yielded similar results across a larger number of assessments. Future studies with larger sample sizes and a greater number of assessments are needed to confirm and extend our findings. Third, our findings may have been influenced by the Hawthorne effect, where participants modify their behaviour due to awareness of being observed, potentially leading to an overestimation of the training’s efficacy [33]. Fourth, we did not test a new training program, but we applied an already validated method by Potgieter et al. [5]. Indeed, our aim was to test the feasibility of training of healthcare practitioners other than specialists, rather to test a new training method. Therefore, even the use of a control group was considered useless for the study aim and we compared our results with a gold standard assessment by an expert tutor. In addition, we recognize that, we intentionally chose a single, immediate training and assessment session to minimize the risk of unequal practice opportunities among participants. In particular, ICU nurses and residents could have had more chances to consolidate their skills during clinical duties compared to medical and nursing students, potentially introducing a relevant bias. While we acknowledge that a staggered, longitudinal ultrasound training approach, together with a frequent technique use, might better support long-term skill retention [34–36], our aim was specifically to evaluate the short-term effectiveness of a standardized training session across different learner groups under controlled conditions. We believe also that future studies could explore the impact of repeated practice over time on skill acquisition and retention in brain ultrasonography, as previously done for other ultrasonographic techniques [34–36]. Lastly, we did not include a long-term follow-up to assess the durability of the training intervention, which remains an important aspect for future research. All these limitations are shared with several previous similar papers [5, 11, 15, 27, 28, 32].

In conclusion, the ONSD technique can be effectively learned by most healthcare providers through a concise 4-h training session, which includes both practical and tutored components. While our findings require further confirmation, they open the possibility for a variety of pre-hospital and intra-hospital use of ONSD, not only by specialized physicians but also by other actors, like well-trained nurses with predefined experience in emergency, urgent, and critical care settings. Further studies assessing the performance of trained operators in critically ill patients, particularly those with pathological ONSD values, will be essential to validate the applicability of our training outcomes in real-world clinical settings. Such investigations should focus not only on measurement accuracy under challenging conditions, but also on the ability of novice operators to reliably detect clinically significant ONSD enlargements, which are more difficult to measure and interpret compared to healthy individuals.

## Supplementary Information

Supplementary Material 1.

## Data Availability

The authors will share all the individual participant data collected during the trial after de-identification, to researchers who provide a methodologically sound proposal. The full protocol and raw data are available at the corresponding authors e-mail address.
