# Correction: Efficacy of a theoretical-practical course for the ultrasound measurement of the optic nerve diameter in different healthcare operators

**DOI:** 10.1186/s13089-025-00435-3

**Published:** 2025-06-30

**Authors:** Eugenio Garofalo, Giuseppe Neri, Vincenzo Bosco, Zaninni Caroleo, Fabiola Virdò, Helenia Mastrangelo, Giusy Guzzi, Gianmaria Cammarota, Chiara Robba, Federico Longhini, Andrea Bruni, ONSD study group, Aldo Mesiti, Antonio Camastra, Antonio Caroleo, Daniele Commisso, Arianna Conidi, Anna Maria Froio, Giuseppe Gaetano, Giuseppe Guerriero, Lucia Lentini, Giuseppe Mazza, Federica Mellace, Silvia Riillo, Giusy Ruocco, Giuseppe Saraco, Deborah Veltri

**Affiliations:** 1 Anesthesia and Intensive Care, Department of Medical and Surgical Sciences, “Magna Graecia” University, Catanzaro, Italy; 2 Department of Neurosurgery, “R Dulbecco” University Hospital, Catanzaro, Italy; 3 Department of Translational Medicine, Eastern Piedmont University, Novara, Italy; 4 Department of Surgical Science and Integrated Diagnostic, University of Genova, Genoa, Italy; 5 Anesthesia and Intensive Care, IRCCS for Oncology and Neuroscience, Policlinico San Martino, Genoa, Italy; 6 Intensive Care Unit, “Mater Domini” University Hospital, Department of Medical and Surgical Sciences, Magna Graecia University, Viale Europa, 88100 Catanzaro, Italy

**Correction: Ultrasound J 17**,** 28 (2025).**

10.1186/s13089-025-00431-7

In this article, the author’s name “Zaninni Caroleo” was incorrectly written as “Caroleo Zaninni”.

The original article [1] has been corrected.
