# Supplementary material for: Efficacy of a theoretical-practical course for the ultrasound measurement of the optic nerve diameter in different healthcare operators
Source: Ultrasound J. 2025 Jun 16;17:28. doi: 10.1186/s13089-025-00431-7 (PMC12170489; doi:10.1186/s13089-025-00431-7)
Supplement: Supplementary file 1 — Supplementary Material 1. [file 13089_2025_431_MOESM1_ESM.docx]

**ELECTRONIC SUPPLEMENTAL MATERIALS**

**EFFICACY OF A THEORETICAL-PRACTICAL COURSE FOR THE ULTRASOUND MEASUREMENT OF THE OPTIC NERVE DIAMETER IN DIFFERENT HEALTHCARE OPERATORS**

Eugenio Garofalo, MD^1^*; Giuseppe Neri, MD^1*^; Vincenzo Bosco, APRN^1^; Caroleo Zaninni, MD^1^; Fabiola Virdò, RN^1^; Helenia Mastrangelo, APRN^1^; Giusy Guzzi, MD^2^; Gianmaria Cammarota, MD, PhD^3^; Chiara Robba, MD^4,5^; Federico Longhini, MD^1^; Andrea Bruni, MD^1^; ONSD study group,

*E.G. and G.N. equally contributed

^1^Anesthesia and Intensive Care, Department of Medical and Surgical Sciences, “Magna Graecia” University, Catanzaro, Italy; ^2^Department of Neurosurgery, “R Dulbecco” University Hospital, Catanzaro, Italy; ^3^Department of Translational Medicine, Eastern Piedmont University, Novara, Italy; ^4^Department of Surgical Science and Integrated Diagnostic, University of Genova, Genoa, Italy; ^5^Anesthesia and Intensive Care, IRCCS for Oncology and Neuroscience, Policlinico San Martino, Genoa, Italy.

**Corresponding author:**

Prof. Federico Longhini, MD

Intensive Care Unit, “Mater Domini” University Hospital,

Department of Medical and Surgical Sciences,

Magna Graecia University, Viale Europa, 88100, Catanzaro, Italy

E-mail: longhini.federico@gmail.com

Tel: +393475395967

**EXPERT CHECKLIST FOR VERIFICATION SESSION**

Trainee ID: _____

🞎 Medical Student 🞎 Nursing Student

🞎 ICU Nurse 🞎 ICU Resident

|  | **#1** | **#2** | **#3** | **#4** | **#5** |
| --- | --- | --- | --- | --- | --- |
| *Correct positioned of the probe on the closed upper eyelid* |  |  |  |  |  |
| *Globe in the center* |  |  |  |  |  |
| *Transverse axis was properly aligned* |  |  |  |  |  |
| *US projection appropriately adjusted* |  |  |  |  |  |
| *Optic nerve at 90°* |  |  |  |  |  |
| *Absenct artifacts or recognized and quality of the imaging improved* |  |  |  |  |  |
| *Measurement was taken perpendicularly* |  |  |  |  |  |
| *Measurement performed 3 mm posterior to the retina* |  |  |  |  |  |
| *Internal ONSD correctly assessed* |  |  |  |  |  |
| *Sensation of pressure on the eye* |  |  |  |  |  |
